# Argon Attenuates Multiorgan Failure in Relation with HMGB1 Inhibition

**DOI:** 10.3390/ijms22063257

**Published:** 2021-03-23

**Authors:** Quentin de Roux, Fanny Lidouren, Agathe Kudela, Lina Slassi, Matthias Kohlhauer, Emilie Boissady, Matthieu Chalopin, Géraldine Farjot, Catherine Billoet, Patrick Bruneval, Bijan Ghaleh, Nicolas Mongardon, Renaud Tissier

**Affiliations:** 1 Service D’anesthésie–Réanimation Chirurgicale, DMU CARE, DHU A–TVB, Hôpitaux Universitaires Henri Mondor, Assistance Publique–Hôpitaux de Paris (AP–HP), F-94010 Créteil, France; quentin.deroux@aphp.fr (Q.d.R.); kudela.agathe@gmail.com (A.K.); nicolas.mongardon@aphp.fr (N.M.); 2 The Mondor Institute for Biomedical Research, Institut National de la Santé et de la Recherche Médicale, University Paris Est Créteil, F-94010 Créteil, France; fanny.lidouren@inserm.fr (F.L.); slassi.lina@gmail.com (L.S.); matthias.kohlhauer@vet-alfort.fr (M.K.); emilie.boissady@vet-alfort.fr (E.B.); bijan.ghaleh@inserm.fr (B.G.); 3 The Mondor Institute for Biomedical Research, Ecole Nationale Vétérinaire d’Alfort, F-94700 Maisons-Alfort, France; 4 Air Liquide Santé International, Centre Innovation Paris, F-78350 Les Loges en Josas, France; matthieu.chalopin@airliquide.com (M.C.); geraldine.farjot@airliquide.com (G.F.); catherine.billoet@airliquide.com (C.B.); 5 UMR 970, Paris Cardiovascular Research Center, INSERM, Hôpital Européen Georges Pompidou, F-75015 Paris, France; patrick.bruneval@aphp.fr

**Keywords:** argon, multiorgan failure, ischemia-reperfusion, inflammation, High Mobility Group Box 1 (HMGB1)

## Abstract

Argon inhalation attenuates multiorgan failure (MOF) after experimental ischemic injury. We hypothesized that this protection could involve decreased High Mobility Group Box 1 (HMGB1) systemic release. We investigated this issue in an animal model of MOF induced by aortic cross-clamping. Anesthetized rabbits were submitted to supra-coeliac aortic cross-clamping for 30 min, followed by 300 min of reperfusion. They were randomly divided into three groups (*n* = 7/group). The Control group inhaled nitrogen (70%) and oxygen (30%). The Argon group was exposed to a mixture of argon (70%) and oxygen (30%). The last group inhaled nitrogen/oxygen (70/30%) with an administration of the HMGB1 inhibitor glycyrrhizin (4 mg/kg i.v.) 5 min before aortic unclamping. At the end of follow-up, cardiac output was significantly higher in Argon and Glycyrrhizin vs. Control (60 ± 4 and 49 ± 4 vs. 33 ± 8 mL/kg/min, respectively). Metabolic acidosis was attenuated in Argon and Glycyrrhizin vs. Control, along with reduced amount of norepinephrine to reverse arterial hypotension. This was associated with reduced interleukin-6 and HMGB1 plasma concentration in Argon and Glycyrrhizin vs. Control. End-organ damages were also attenuated in the liver and kidney in Argon and Glycyrrhizin vs. Control, respectively. Argon inhalation reduced HMGB1 blood level after experimental aortic cross-clamping and provided similar benefits to direct HMGB1 inhibition.

## 1. Introduction

Multiorgan failure (MOF) is a syndrome encountered in various clinical settings such as shock and ischemia-reperfusion injuries, generating major medical, ethic and economic issues [1]. Its management is mainly limited to pharmacological or mechanical organ support, along with etiological treatment. Alternative strategies are thus being eagerly investigated. Among them, inhalation of noble gases such as xenon or argon could provide organo-protective effects [2,3]. If xenon provides interesting neuroprotective effects, its costs and rarity limit its use and led to the surge of interest in argon [4]. This more abundant gas extracted from air exerts anti-ischemic effects and provides promising experimental results in animal studies. These benefits have been mainly studied after regional ischemia with “single organ” dysfunction or after cardiac arrest for neuroprotective purpose [5,6,7,8,9,10]. Multiorgan protection was also reported by our group in a model of MOF induced by supra-coeliac aorta cross-clamping in rabbits [2]. 

Mechanistically, argon has been shown to increase the resistance to ischemic injuries through direct cytoprotective effects involving the extracellular signal-regulated kinases (ERK ½) pathway [11]. However, other studies have reported anti-inflammatory effects with a reduction in interleukins (IL)-6 and IL-1β release, as well as reduced Toll-like receptor 2 and 4 density on cell surface in vitro [12,13,14]. Yet, the links between the anti-inflammatory and anti-ischemic properties are not established. Determining whether argon could provide specific benefits for MOF prevention with multiple cross-talks between end-organ injuries is thus crucial [3,11]. In the present study, we hypothesized that this link could actually involve High Mobility Group Box 1 (HMBG1), since this well-known activator of innate immunity is released by ischemic cell death and has been shown to be reduced after argon exposure in a rat cardioplegia model [15,16]. 

Accordingly, the aim of this study was to investigate whether argon-induced attenuation during MOF is associated with HMGB1 release inhibition and provides similar benefits than the direct pharmacological inhibition of HMGB1. For the latter purpose, we used the well-known HMGB1 inhibitor glycyrrhizin [17,18,19]. We used a model of MOF through aortic cross-clamping in rabbits, that allowed us to investigate cardiovascular, intestinal, hepatic and renal injuries, as well as blood inflammatory biomarkers. 

## 2. Results

### 2.1. Hemodynamic Parameters

**Figure 1:**
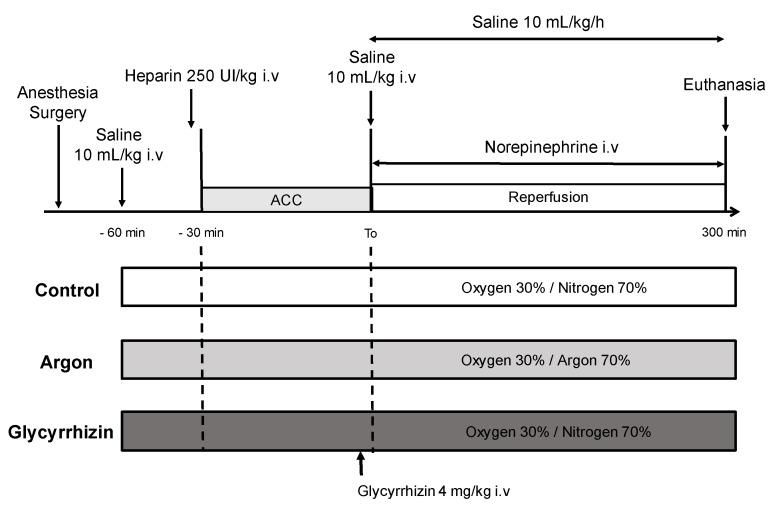
Schematic representation of the experimental protocol. ACC: aortic cross-clamping; Control (*n = 7*), Argon (*n* = 7), Glycyrrhizin (*n* = 7).

**Figure 2:**
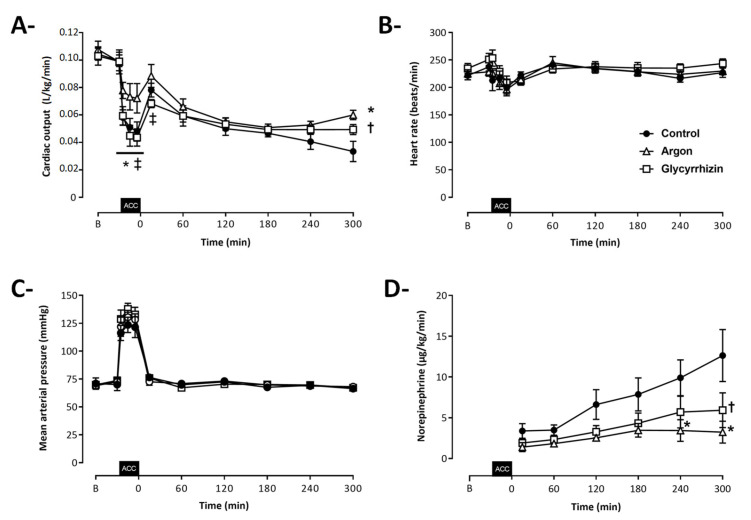
Cardiac output (**A**), heart rate (**B**), mean arterial pressure (**C**) and norepinephrine regimen of administration (**D**) throughout protocol. ACC: Aortic cross clamping; * *p* < 0.05 between Control and Argon; ^†^ *p* < 0.05 between Control and Glycyrrhizin; ^‡^ *p* < 0.05 between Argon and Glycyrrhizin; Control (*n* = 7), Argon (*n* = 7), Glycyrrhizin (*n* = 7).

Seven rabbits were included in each experimental group (Figure 1). As illustrated by Figure 2, cardiac output, heart rate and mean arterial pressure were similar among groups before aortic cross-clamping. During aortic cross-clamping, cardiac output was significantly higher in the Argon as compared to the Control and Glycyrrhizin groups. After aortic cross-clamping, a gradual decrease in cardiac output was further observed in all groups, with higher values in Argon and Glycyrrhizin groups at the end of the follow-up, as compared to Control (60 ± 4, 49 ± 4 and 33 ± 8 mL/min/kg, respectively; Figure 2A; *p* = 0.0002 between Argon and Control and *p* = 0.0213 between Glycyrrhizin and Control). This was not related to any differences in heart rate, which were similar among groups throughout follow-up (Figure 2B). Mean arterial pressure was also not significantly different between groups (Figure 2C) but norepinephrine requirements were reduced in Argon and Glycyrrhizin groups as compared to Control (3.2 ± 1.3, 5.9 ± 2.1 and 12.6 ± 3.0 μg/kg/min at the end of the follow-up, respectively; Figure 2D).

### 2.2. Biochemical Alterations of Shock

**Figure 3:**
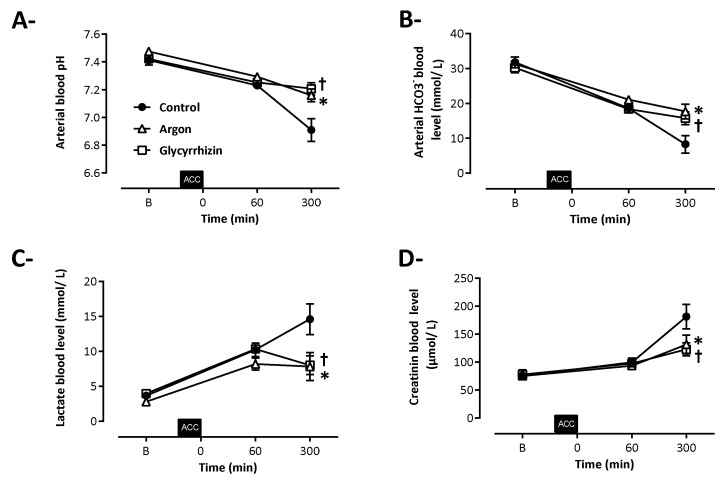
Arterial pH (**A**) and blood levels of HCO_3_– (**B**), arterial lactate (**C**) and plasma creatinine (**D**). ACC: Aortic cross clamping; * *p* < 0.05 between Control and Argon; † *p* < 0.05 between Control and Glycyrrhizin; Control (*n* = 7), Argon (*n* = 7), Glycyrrhizin (*n* = 7).

As shown in Figure 3, biochemical hallmarks of shock were evidenced in all animals. In the Control group, aortic cross-clamping resulted in severe metabolic acidosis and renal injury. In comparison, arterial pH was significantly higher at the end of the follow-up in Argon and Glycyrrhizin groups vs Control (7.16 ± 0.06, 7.21 ± 0.06 and 6.91 ± 0.09, respectively; Figure 3A; *p* = 0.0003 between Argon and Control and *p* < 0.0001 between Glycyrrhizin and Control), along with higher plasma bicarbonate concentrations (17.7 ± 2.8, 15.8 ± 2.3 and 8.3 ± 2.7 mmol/L, respectively; Figure 3B; *p* = 0.0002 between Argon and Control and *p* = 0.0025 between Glycyrrhizin and Control), lower lactate concentrations (7.8 ± 2.6, 8.0 ± 2.2 and 14.6 ± 2.4 mmol/L, respectively; Figure 3C; *p* = 0.0019 between Argon and Control and *p* = 0.0025 between Glycyrrhizin and Control) and reduced creatinine blood levels (132 ± 22, 123 ± 15 and 181 ± 24 µmol/L, respectively; Figure 3D; *p* = 0.0038 between Argon and Control and *p* = 0.0126 between Glycyrrhizin and Control).

### 2.3. Cytokine Blood Levels

**Figure 4:**
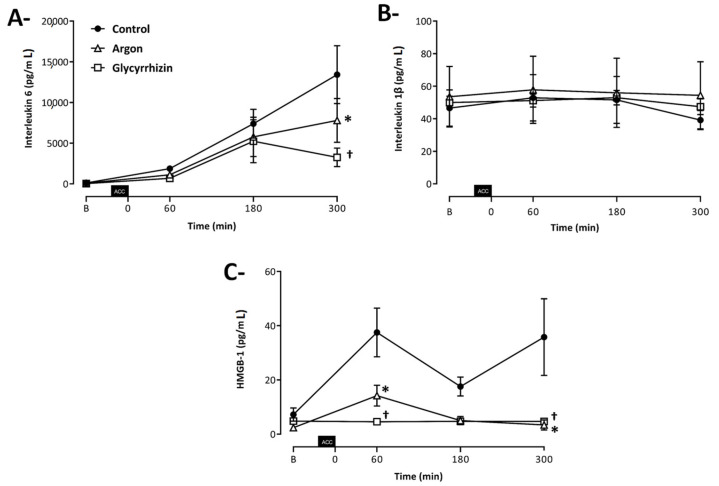
Blood levels of interleukin 6 (**A**), interleukin 1β (**B**) and High Mobility Group Box 1 (**C**). ACC: Aortic cross clamping; * *p* < 0.05 between Control and Argon; † *p* < 0.05 between Control and Glycyrrhizin; Control (*n* = 7), Argon (*n* = 7), Glycyrrhizin (*n* = 7).

As illustrated in Figure 4A, a progressive and large increase in blood IL-6 concentrations was observed after unclamping in the Control group, with a significant reduction in Argon and Glycyrrhizin groups vs Control (7504 ± 2481, 3251 ± 1229 and 13420 ± 383 pg/mL, respectively; *p* = 0.0296 between Argon and Control and *p* < 0.0001 between Glycyrrhizin and Control). Conversely, blood IL-1β concentrations were not modified during follow-up and among groups (47.4 ± 12.8, 39.4 ± 14.4 and 54.4 ± 50.5 pg/mL at the end of follow-up; Figure 4B; *p* = 0.4273 between Argon and Control and *p* = 0.6536 between Glycyrrhizin and Control). 

At baseline, blood HMGB1 concentration did not differ between groups. After 60 min of reperfusion, an early and significant decrease in blood HMGB1 concentration was observed in Argon and Glycyrrhizin groups vs Control (14.2 ± 4.2, 4.5 ± 0.1 and 37.5 ± 9.7 pg/mL, respectively; *p* = 0.0021 between Argon and Control and *p* < 0.0001 between Glycyrrhizin and Control). At 300 min of reperfusion, blood HMGB1 concentration was still significantly lower in Argon and Glycyrrhizin groups, while a secondary increase was observed in Control (3.4 ± 1.9 and 4.7 ± 0.1 vs. 35.8 ± 14.1 pg/mL, respectively; Figure 4C; *p* < 0.0001 between Argon and Control and *p* < 0.0001 between Glycyrrhizin and Control). 

### 2.4. Histopathological Evaluation

**Figure 5:**
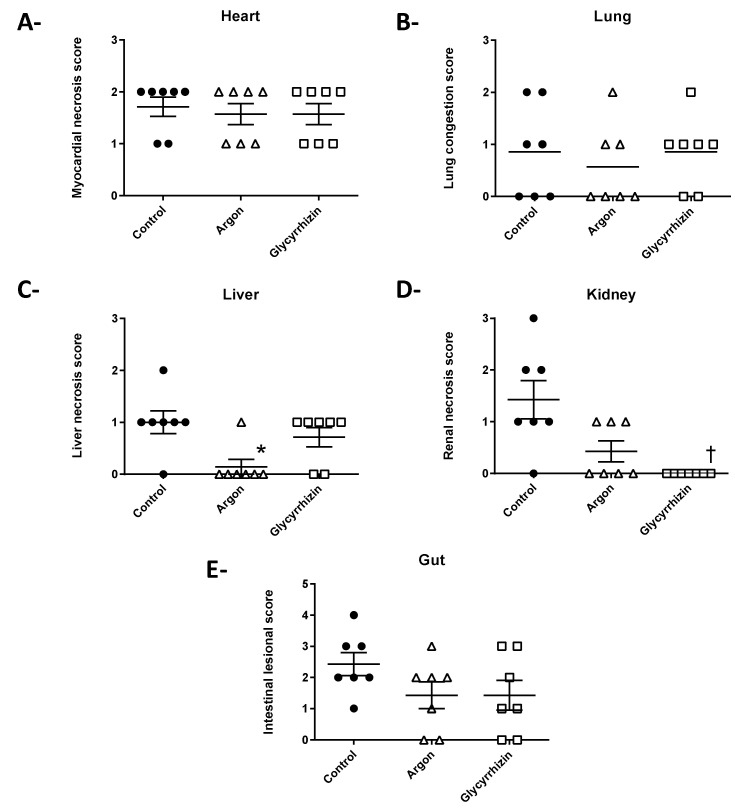
Histopathological score of alteration in the heart (**A**), lung (**B**), liver (**C**), kidney (**D**) and gut (**E**). * *p* < 0.05 between Control and Argon; † *p* < 0.05 between Control and Glycyrrhizin.

Histopathological lesions revealed ischemic lesions in most organs (score > 0). In myocardium and pulmonary samples (Figure 5A,B), myocardial necrosis and lung congestion lesions were similar in all groups. In contrast, tubular necrosis lesions were less pronounced in Argon group (without significant difference) but significantly lower in the Glycyrrhizin group, as compared to Control (Figure 5C). Conversely, liver lesions of centrilobular clarification were significantly attenuated with Argon but not Glycyrrhizin, as compared to Control (Figure 5D). No significant difference was observed on intestinal necrosis lesions (Figure 5E).

### 2.5. Immunohistochemistry

**Figure 6:**
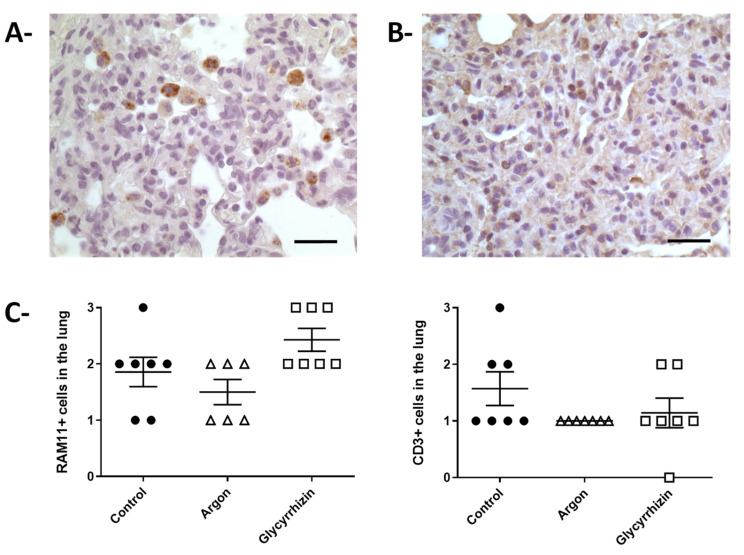
Immunochemistry in lung parenchyma. (**A**)—Morphological appearance of the immunohistochemical staining for macrophage identification using the RAM11 polyclonal antibody (bar = 25 µm). (**B**)—Morphological appearance of the immunohistochemical staining for T cells identification using a CD3 antibody (bar = 25 µm). (**C**)—Semiquantification of RAM11 and CD3 positive cells in the pulmonary samples from the different groups.

Since the pathologist observed a strong inflammation during the blind evaluation of the lung samples, we performed immunohistochemical analysis for the evaluation of macrophages (RAM11 + cells) or T cells (CD3+ cells) in pulmonary parenchyma (Figure 6). No difference was observed regarding lung infiltration with RAM11+ cells (macrophages) among groups. A non-significant increase was observed in Glycyrrhizin group as compared to Argon and Control. Conversely, a trend toward a reduction in CD3+ cells was observed in both Argon and Glycyrrhizin groups vs Control.

## 3. Discussion

In the present study, argon inhalation and HMGB1 inhibition by glycyrrhizin attenuated MOF induced by aortic cross-clamping. This situation mimics various medical or postoperative scenes encountered in critically ill patients. The benefits were observed on cardiac output, norepinephrine requirements, metabolic acidosis, and renal or hepatic damages. In the Argon group, cardiovascular benefits followed a multi-phasic pattern of improvements, with an initial improvement in cardiac output during aortic cross-clamping, and then a deterioration with an ultimate re-increase at the end of the follow-up. In the Glycyrrhizin group, cardiac output was only improved at the end of the follow-up vs. Control. In both groups, blood levels of HMGB1 and IL-6 were significantly reduced, as compared to Control. 

The present results reinforce our previous observations with argon alone in similar experimental conditions in rabbits [2]. In this previous study, argon inhalation reduced hemodynamic alterations with maximal benefits when inhaled throughout aortic cross-clamping and unclamping as compared to post-clamping only. Opposite to the present study, we did not observe any increase in cardiac output with argon during aortic cross-clamping as compared to Control, but only after unclamping. During the previous study, the mechanical ventilation led to a positive end-expiratory pressure (~3–5 cmH_2_O), which was not the case in the current experiment with zero end-expiratory pressure. This difference could have hampered the vasodilatory effect of argon on pulmonary vessels, which might contribute to the cardiac output increase in the present study [20]. Obviously, this is still only a hypothesis that deserves further investigation but that could open perspectives for argon use during cardiogenic shock. In the same line, the recent study of Fumagalli et al. in a porcine model of cardiac arrest also reported hemodynamic benefits with argon after resuscitation [9].

More importantly, the present study shows that argon reduced HMGB1 blood levels while the direct inhibition of this protein with glycyrrhizin also provided benefits. Then, argon could exert an initial direct anti-ischemic effect that reduces the passive release of the nuclear HMGB1. Several studies have indeed shown that argon could exert, for instance, direct anti-apoptotic effects through caspase-3 inhibition or modulation of the Bcl2 pathway [13,21]. Argon was also shown to increase extracellular signal-regulated kinase (ERK ½) phosphorylation, which increases cell resistance to ischemic damages [6,14]. These effects could reduce the initial drop in HMGB1 blood levels, which could explain the later anti-inflammatory effects of argon on IL-6 blood levels [16].

Indeed, the use of the direct HMGB1 inhibitor glycyrrhizin just before aortic cross-unclamping reduced HMGB1 and IL-6 blood levels with a similar magnitude than argon. This licorice-derived glycoside is known to act through a direct binding to HMGB1, which reduced subsequent cytokine production [22]. For instance, it was shown to provide neuroprotection after spinal cord ischemia-reperfusion injury or cardioprotection after myocardial infarction model [18,23]. Since glycyrrhizin completely blunted HMGB1 blood concentration increase after unclamping, this protein is probably promoting a proper vicious circle after aortic cross-clamping with an initial minor release due to ischemia and then a continuous active secretion promoted by the protein itself on immune cells. Similar auto-promotion of HMGB1 release was indeed well demonstrated in other conditions and could perpetuate the inflammatory reactions during MOF or after ischemic injuries [24]. Then, argon might exert its benefit through an initial reduction of HMGB1 by pure anti-ischemic effect and then lead to secondary benefits with the subsequent blunting of HMGB1 inflammatory vicious circle. It is supported by our previous study showing that argon administration before and just after aortic cross-clamping prevented MOF with a magnitude close to that afforded by argon inhalation throughout the ischemic and reperfusion period [2]. It could be important since it suggests that argon may not directly alter the inflammatory reaction, but rather exerts pure anti-ischemic effect that might just attenuate the subsequent innate immunity activation. As shown by the lung immunohistochemical analyses, this attenuation did not blunt lung immune cells infiltration, even if a trend toward less T cell infiltration was importantly observed. 

This study has several limitations. First, it was not possible to directly address the link between argon and HMGB1, since it would require a direct activation of HMGB1 pathway on top of argon, expecting a loss of protection. We would need analogs of HMGB1 which are activating its receptors, such as the receptor for advanced glycation end products (RAGE), while HMGB1 is known to exert more complex effects, depending upon its redox status and multiple receptors [24]. The direct activation of RAGE would then lead to much stronger inflammatory reaction with multiple deleterious effects that could limit the relevance of this experiment. We then chose to compare argon and glycyrrhizin, with a concomitant evaluation of HMGB1 blood level. Second, glycyrrhizin is acting through HMGB1 inhibition but might also exert other pharmacological effects on mineralocorticoid pathways [25]. However, if the latter effect is known to participate in the hypertensive effect of glycyrrhizin after chronic exposure, it is believed to be irrelevant after a single exposure, explaining why this molecule is typically used for HMGB1 mitigation [26]. In addition, we conducted preliminary experiments that confirmed that glycyrrhizin did not exert any proper hemodynamic effects in sham animals (data not shown). Finally, one would be surprised by the large increase in IL-6 blood levels in our conditions, while IL-1β blood levels were not modified. In a previous report after generalized ischemia, we indeed also observed that IL-6 was activated very early, whereas IL-1β or tumor necrosis factor-alpha were not [27]. An increase in IL-6 has been also reported in swine undergoing infra-renal clamping [28]. Conversely, argon did not significantly modify mRNA blood levels of several cytokines in a previous study in similar conditions, suggesting that argon only reduced reactivity of inflammatory cells through reduced HMGB1 levels but not transcription levels. However, we only investigated a few markers and did not decipher the immune response after aortic cross-clamping, which was not the goal of the study. More importantly, argon could also likely modify other pro-inflammatory markers of cell injury even if we here focused on HMGB1.

## 4. Materials and Methods

All experiments were conducted in compliance with the French legislation governing animal research. The experimental procedure was approved by the institutional review board for animal research (APAFIS#17800–2020012017214953, Cometh Anses-EnvA-UPEC n°16). 

### 4.1. Animal Preparation 

As previously described [2,29], male New Zealand rabbits (2.0–3.0 kg) were anesthetized using a mixture of zolazepam and tiletamine (12.5 mg/kg i.v), thiopental (10 mg/kg i.v) and methadone chlorhydrate (0.6 mg/kg i.v). After tracheal intubation, mechanical ventilation was started with inspired fraction of oxygen set at 30%, tidal volume at 10 mL/kg and respiratory rate at 28–30 cycles/minute. Electrocardiogram was continuously monitored, as well as blood pressure through a pressure catheter inserted into the right carotid artery. A rectal temperature probe was inserted for body temperature control at 38.0 ± 0.5 °C with heating pads. After left thoracotomy, a 4 mm diameter flow probe was placed around the ascending aorta to continuously measure cardiac output (PS–Series Probes, Transonic, NY, USA). Then, a left laparotomy incision was performed to isolate the abdominal supra-coeliac aorta. After instrumentation, saline (NaCl 0.9%, 10 mL/kg i.v) and heparin (250 IU/kg i.v) were administered. Bladder was emptied before aortic cross-clamping to measure urinary output.

### 4.2. Protocol of Aortic Cross-Clamping 

After a period of stabilization, supra-coeliac aorta was occluded with a vascular clamp. The occlusion was released after 30 min of aortic cross-clamping, as previously described [2,28]. To optimize hemodynamics, additional administration of saline (10 mL/kg i.v) was performed just after unclamping, followed by a continuous infusion (10 mL/kg/h i.v) until the end of experiment. Norepinephrine administration was adjusted to target a mean arterial pressure of 70 mmHg. Animals were then monitored for 300 min of reperfusion after aortic unclamping. Then, they were euthanized using a lethal dose of pentobarbital (60 mg.kg–1 i.v). Lungs, heart, liver, gut and kidneys were sampled and fixed in formaldehyde (4%) for blinded histological analysis.

### 4.3. Experimental Protocol

Prior to aortic cross-clamping, the animals were assigned to one of the 3 experimental groups using a block randomization (Figure 1). In the Control group, animals underwent conventional mechanical ventilation with a mixture of nitrogen (inspired fraction 70%) and oxygen (30%). In the Argon group, animals inhaled mixture of argon (70%) and oxygen (30%). Argon inhalation started 30 min before aortic cross-clamping and was maintained until the end of the protocol, i.e., 300 min after reperfusion. In the Glycyrrhizin group, mechanical ventilation was performed with nitrogen/oxygen mixture (70%/30%) with an administration of glycyrrhizin (4 mg/kg i.v) five minutes before reperfusion [30]. The investigator taking care of the animals was not blinded to the treatment group.

### 4.4. Immuno–Assay and Histology

Plasma concentrations of IL-6, IL1-β and HMBG1 were evaluated by enzyme-linked immunosorbent assay (IL-6 and IL1-β: R&DSystems^®^, Minneapolis, MN, USA); HMBG1: Abbexa^®^, Cambridge, UK). Heart, lungs, kidneys, liver and gut were submitted to blinded histopathological analyses after haematoxylin–eosine–safran staining. For the heart, kidney and liver, ischemic injury was blindly graded using a score from 0 (normal tissue) to 3 (extensive cell necrosis). For the lung, a score of lung congestion and alveolar edema was attributed from 0 (no congestion and edema) to 3 (severe congestion and serous alveolar edema). Finally, intestinal damages were graded using a modified Chiu score from 0 (normal) to 5 (severely damaged), as previously described [2].

Since we observed lung inflammation during histopathological evaluation, immunohistochemical analyses were further performed to quantify the invasion of the pulmonary parenchyma by immune cells. T-cells were detected with a monoclonal antibody against a CD3 antigen (Orb323391, BIORBYT, Cliniscience, Nanterre, France). Macrophages were detected with a monoclonal antibody (RAM11, VWR, Fontenay–sous–Bois, France). Cell infiltration was blindly graded from 0 to 3. 

### 4.5. Statistical Analysis

Data are expressed as mean ± SEM for continuous parameters and individual values and medians for histological scores, respectively. The primary outcome was the cardiac output at the end of the follow-up. Secondary outcomes were other hemodynamic, biochemical and histopathological parameters. Hemodynamic parameters were compared between groups by a two-way analysis of variance (ANOVA) for repeated measurements at baseline, during aortic cross-clamping (at 5, 15 and 25 min) and during reperfusion (at 60, 120, 180, 240 and 300 min). If necessary, a post-hoc Holm–Sidak test was performed for comparison to Control value at each time (no between-time comparison). Biochemical parameters (except cytokines) were compared between groups by the same statistical analysis, except for the baseline value, where a non-parametric Student’s test was performed (one time only). Inflammatory markers were compared with a Kruskall–Wallis analysis followed if necessary by a Mann–Whitney test. Significant differences were determined at *p* ≤ 0.05.

## 5. Conclusions

Both argon and glycyrrhizin attenuated MOF in a rabbit model of aortic cross-clamping, leading to improved cardiac output and attenuated shock and renal and hepatic ischemic lesions. The action of argon could involve multiple mechanisms including direct hemodynamic benefits during aortic cross-clamping, reduced cell death at reperfusion and attenuation of damage perpetuation through HMGB1 release initial inhibition.

## 6. Patents

Quentin de Roux, Matthieu Chalopin, Géraldine Farjot, Catherine Billoet, Nicolas Mongardon and Renaud Tissier are named as co-inventors on a patent application related to the use of inhaled argon in patients with cardiovascular diseases.

## Data Availability

The data presented in this study are available on request from the corresponding author.
